# Functional Properties of Glutinous Rice Flour by Dry-Heat Treatment

**DOI:** 10.1371/journal.pone.0160371

**Published:** 2016-08-18

**Authors:** Yang Qin, Chengzhen Liu, Suisui Jiang, Jinmiao Cao, Liu Xiong, Qingjie Sun

**Affiliations:** College of Food Science and Engineering, Qingdao Agricultural University, Qingdao, Shandong Province, 266109, China; Tennessee State University, UNITED STATES

## Abstract

Glutinous rice flour (GRF) and glutinous rice starch (GRS) were modified by dry-heat treatment and their rheological, thermal properties and freeze-thaw stability were evaluated. Compared with the native GRF and GRS, the water-holding ability of modified GRF and GRS were enhanced. Both the onset and peak temperatures of the modified samples increased while the endothermic enthalpy change decreased significantly (*p* < 0.05). Meanwhile, dry heating remarkably increased the apparent viscosities of both GRF and GRS. Importantly, compared with GRS samples, the storage modulus (G') and loss modulus (G") values of modified GRF increased more greatly and the tanδ values decreased more remarkably, indicating that the dry-heat treatment showed more impact on the GRF and a higher viscoelasticity compared with GRS. Our results suggest the dry-heat treatment of GRF is a more effective method than that of GRS, which omits the complex and tedious process for purifying GRS, and thereby has more practical applications in the food industry.

## Introduction

Rice starch is one of the important commercial cereal starches. Because of the unique physiochemical properties (such as small granules, low allergenicity, and increased freeze—thaw stability of pastes), rice starch has been applied as cosmetic dusting powder, photographic paper powder, food thickener, and excipient for pharmaceutical tablets [1–2]. However, the close conjunction between the starch granules and surrounding protein matrix results in difficult isolation of rice starch. Currently, glutinous rice flour (GRF) has been widely used in both novel and traditional foods such as sweet soup balls, infant foods, puffed grains and gluten free products [3–4] due to the soft, high sticky nature and easily digestible carbohydrates after cooking [5]. More importantly, GRF is rich in protein, mineral substances, and vitamins, and is more nutritious than glutinous rice starch (GRS). However, GRF has negative aspects as well as GRS, such as poor resistance to shear force, and low elastic gel-forming ability, leading to its limited application in food industry. Therefore, it is necessary to enhance its inherent properties in accordance with the intended purposes in the products. Rice flour can be modified in several ways, such as chemical, physical, and enzymatic methods [6–7]. Physical modifications involve pre-gelatinization and heat-moisture treatment [8–9], and have gained a wider application [3, 10].

Dry heating is one newly developed physical modification method to produce modified starches, which is simple, safe, and produces no pollution. In recent years, much attention has been paid to the effect of dry heating on the pasting and thermal properties of starch and starch with hydrocolloid [11]. Starch’s functional properties, such as oil-binding capacity and water-binding capacity of potato, sweet potato, and taro starches, increased significantly when modified with ionic gums and dry heating [12]. Li et al. [13] studied that after dry-heat treatment, the gel-forming ability of waxy rice starch with xanthan was strengthened, as both storage and loss modulus values increased. Besides, dry heating with sodium alginate or carboxymethyl cellulose (CMC) would enhance the paste viscosity of waxy maize starch [14]. Sun et al. [15] also found that the gel structure of potato starch became more compact after dry heating with CMC. In addition to starch, protein is the second most abundant biomass components in the flour. Falade and Onyeoziri [16] have demonstrated that the peak viscosities of yam flour after dry-heat treatment significantly increased. Sun et al. [11] reported that dry-heat treatment had a more significant influence on the thermal properties of flour than of starch, and the difference may be attributed to the existence of the non-starch components such as protein in the flour. Dry heating on morphological, structural, and pasting properties of GRS and GRF was studied by Qiu et al. [17], who found that the crystallinity of modified GRS and GRF were increased, and the morphological structures of modified GRF were denser than that of modified GRS.

Because of the freeze—thaw stability is one of the key determinants for starches used as the clean-label ingredients in frozen food products, and the rheological properties could adequately reflect the viscoelasticity and stability of the GRF and GRS gels, which would help us better understand the importance of the viscoelastic properties of GRF and GRS suspension utilized in the fields of food industry. Although the rheology and freeze-thaw stability of different types of starches or flours via physical modification methods such as pregelatinization and heat-moisture treatment have been investigated in previous literatures [8–10], for dry-heat treatment, as a new physical modification method, no detailed information (containing our previous article) can be obtained about its effect on the rheological, freeze-thaw stability and thermal properties of GRF and GRS. To fill this knowledge gap, we aimed to investigative the differences in the rheological properties and freeze-thaw stability between the modified GRF and GRS via dry heating for their better application in food industries especially in high viscosity food, and then to make further evaluate whether dry heated GRF can be used as a substitute for GRS in food industry.

## Materials and Methods

### 2.1 Materials

Glutinous rice grains (Longnuo 3) were obtained from the Rice Research Institute, Heilongjiang Academy of Agricultural Sciences (Heilongjiang, China). Analytical grade chemicals were supplied by Tianjin Jiangtian Chemical Co. Ltd. (Tianjin, China).

### 2.2 Preparation of glutinous rice flour and starch

The rice grains were ground into flour using cryogenic milling as described by Hasjima et al. [18] with some modification. Rice grains (1,000 g) were steeped in 2 times volume deionized water container which placed in a refrigerator (Haier BCD-225SLDA, Qingdao, China) at 4°C for 2 h, then the water was drained off and put into a high speed blender (FW100, Tianjin, China) which grinded the grains into powders. GRF within the retort pouch was dried in a hot air oven at 45°C until the moisture content reduced to 7.0%, and then the dried GRF passed through a 100-mesh screen.

Glutinous rice starch was prepared using the alkaline steeping method [19] with some modifications. Rice grains (1,000 g) were soaked in 2 times volume 0.35% (w/v) sodium hydroxide solution at 4°C for 24 h. The supernatant was drained off and the rice grains were ground with a blender, and then passed through a 100-mesh screen. The slurry was centrifuged at 3,000 rpm for 15 min, the supernatant was decanted off, and the starch layer was re-slurred with triple-volume deionized water. The step of washing the starch layer with deionized water was applied four times. Subsequently, the starch was neutralized with 1 N hydrochloric acid to pH 7. Precipitated starch was collected by centrifugation at 3,000 rpm for 15 min, the supernatant was removed. Afterward, the starch cakes was dried in a hot air oven at 45°C for 24 h until the moisture content reduced to 7.0%. The moisture content was measured by a Sartorius AG moisture meter (MA-45). The dried GRS was ground to pass through a 100-mesh sieve.

### 2.3 Dry-heat treatments of glutinous rice flour and starch

The flours and starches were modified by dry-heat treatment according to Lim et al. [14], with some modifications. The GRF (7.0%, w/w, moisture content) was also heated for 0, 2, and 4 h at 130°C (GRF, GRF2 h, GRF4 h) in the oven (876A-2, Shanghai, China), and GRS (7.0%, w/w, moisture content) was heated for 0, 2, and 4 h at 130°C (GRS, GRS2 h, GRS4 h), respectively. After dry-heat treatment, the treated samples were cooled to the room temperature and then stored in 50 mL plastic tubes with caps to prevent them from being affected by damp conditions for further analysis. Untreated GRF and GRS were used as controls.

### 2.4 Determination of chemical compositions of glutinous rice flour and starch

The proximate compositions of GRF and GRS were analyzed according to AOAC methods [20] for the determination of moisture, ash, protein, and crude fat contents. The protein content of the rice flour samples was determined by the Kjeldahl method [21]. The result was multiplied by the factor 5.95 to convert to crude protein content.

### 2.5 Differential scanning calorimetric (DSC) measurement

Thermal parameters of all the samples were measured using a differential scanning calorimeter (DSC1; Mettler Toledo, Schwerzenbach, Switzerland) equipped with a thermal analysis data station and data recording software (STAR@ SW 9.20), as described by Ahmed et al. [22]. Each sample (approx. 4 mg) and distilled water (8 mg) were placed in an aluminum pan, then sealed in the aluminum hermetic pan and then kept at 4°C for 24 h. The scanning temperature range and the heating rates were 25–120°C and 10°C/min, respectively. In all measurements, the thermogram was recorded with an empty aluminum pan as a reference. During the scans, the space surrounding the sample chamber was flushed with dry nitrogen to avoid condensation. The transition temperatures reported are the onset (To), peak (Tp), conclusion (Tc) and gelatinization temperature range (Tc−To). The gelatinization enthalpy change (ΔH) estimated by integrating the area between the thermogram and a baseline under the peak, was expressed in Joules per gram of dry basis sample.

### 2.6 Freeze-thaw stability

Syneresis of freeze-thawed GRF and GRS gels were determined as described by Sun and Yoo [23], with minor modifications. An aqueous suspension of sample (5 g/100 g) was heated at 95°C under constant mild agitation for 30 min and then cooled to room temperature in an ice water bath. The paste was weighed (15 g) in centrifuge tubes and subjected to successive freeze-thaw cycles by freezing at –18°C for 24 h and thawing at 50°C for 30 min, followed by centrifugation at 3,000 × g for 15 min. The supernatant eliminated from the gel was weighed, and the extent of syneresis was expressed as the percentage of liquid separated per total weight of sample in the centrifuge tube. The syneresis percentage was calculated using Eq 1:

Syneresis(%)=Ws/W×100 (1)

where Ws is the weight of the water separated from the gels, and W is the weight of the gel.

### 2.7 Measurement of static rheological parameter

GRF and GRS suspensions (12%, w/v) were prepared by adding the powders (3 g) to distilled water (25 mL) in an aluminum can (36 mm diameter). The suspensions were gelatinized via a rapid viscosity analyzer (RVA, Model 4D, Newport Scientific, Australia) according to the methodology by Sun et al. [15]. The obtained hot pastes were immediately transferred to the platen of a rheometer (MCR102, Anton Paar, Austria), which was equipped with a smooth parallel plate measuring geometry (50 mm diameter, 1° cone angle) at the gap size 1 mm. The continuous shear tests were performed at 25°C over the shear rate range of 0.01–300 s^-1^ to measure the influence of the shear rate on the apparent viscosity and shear stress, as well as to describe the steady shear rheological properties of the samples, and the data were fitted to the well-known the power law model of Herschel-Bulkley (Eq 2):

$$\tau =\tau_{0}+K\cdot \gamma^{n}$$ (2)

Where τ = shear stress (Pa), τ_0_ = yield stress (Pa), K = consistency coefficient (Pa·s^n^), γ = shear rate (s^−1^), and n = flow behavior index. Furthermore, n is the flow behavior index, which demonstrates the extent to which the liquid departs from Newtonian fluid.

### 2.8 Measurement of dynamic rheological parameter

The dynamic rheological properties of GRF and GRS were measured (MCR102, Anton Paar, Austria) according to previous literature by Sopadea et al. [24]. The freshly prepared hot sample pates (12%, w/v) from RVA were put into the testing platform of the dynamic rheometer, and were added to the peltier plate, and the parallel plate geometry (50 mm) at gap 1 mm. After removing the excess suspension and placing silica oil to the edge of the plate, the frequency sweeps were performed at 25°C over the angular frequency range of 0.1–70 rad/s. The 0.5% strain was in the linear viscoelastic region according to the strain sweep results (data not shown) with the frequency of 1 Hz. The mechanical spectra were obtained to record the storage modulus (G'), loss modulus (G"), and loss tangent (tanδ = G"/ G') as a function of the frequency (ω) determined in triplicate.

### 2.9 Statistical analysis

All experiments were conducted at least three times, and then experimental data were analyzed using an analysis of variance (ANOVA) and were expressed as mean values ± standard deviations. Differences were considered at a significance level of 95% (*p* < 0.05). Pearson’s correlation coefficients among parameters were calculated using the Statistical Package for the Social Sciences (SPSS) v 17.0 software.

## Results and Discussion

### 3.1 Chemical composition

The chemical compositions of GRF and GRS samples are presented in Table 1. GRS had much higher contents of total starch (91.78%) compared to GRF (83.59%). GRF contained noticeably higher amounts of protein and lipid compared to rice starch. Protein, lipid, and ash in GRF were 7.78%, 0.97%, and 0.45% (dwb, %), whereas those in GRS were 0.49%, 0.30%, and 0.26% (dwb, %), respectively. Previous studies have shown that rice starch contained 0.07–0.68% protein and 0.01–0.35% ash [25] whereas rice flour contained 71–91% starch, 7–11% protein, 0.87–8.10% lipid and 0.46–1.10% ash [25–26]. Similarly, Falade and Christopher [27] found that the fat and protein contents of rice starches varied from 0.04 to 0.35 and 0.35–0.48%, respectively. Differences in the chemical compositions of GRS and GRF would influence their thermal and gel properties, as well as the susceptibility of flour and starch to dry-heat treatment.

**Table 1:** Chemical Composition of glutinous rice starch (GRS) and glutinous rice flour (GRF).

Sample	Starch (%)	Protein (%)	Lipid (%)	Moisture content (%)
GRS	99.32±0.76^a^	0.49±0.01^b^	0.03±0.01^b^	7.01±0.13^a^
GRF	87.23±0.74^b^	6.78±0.17^a^	0.71±0.12^a^	7.21±0.34^a^

Values of means followed by different lowercase letters in the same column are significantly different (*p* < 0.05). Values expressed are means ± standard deviations (n = 3).

### 3.2 Thermal properties

The thermal parameters of native and modified GRF and GRS are given in Table 2. The onset temperature (To) of native GRS was 59.08°C, which was in accordance with Zhu et al. [25] who reported the To of the rice starches ranged from 58.80 to 70.40°C. After dry-heat treatment, the gelatinization endotherms of the modified starch and flour shifted to a higher temperature with a prolongation of dry-heat treatment time. The peak temperature (Tp) of flour had the highest increase of about 4.7°C after dry-heat treatment, which was from 63.07°C (GRF) to 65.09°C (GRF2 h) and 67.79°C (GRF4 h). Moreover, Chen et al. [28] reported that the increase in the gelatinization temperature of modified starch and flour has been attributed to amylose-amylose, amylose-amylopectin interactions, as well as chemical bonding/interactions that occur during heat-moisture treatment. However, the gelatinization temperature range (Tc−To) of dry heated GRF was significantly (*p* < 0.05) different from those of dry heated GRS treated under the same conditions. The Tc−To of GRF were 17.28°C, 15.31°C, and 13.10°C, whereas those in GRS were 13.01°C, 12.68°C, and 11.21°C, respectively. The Tc−To of native flour (17.28°C) was higher than that of native starch (13.01°C), indicating that other components besides starch in the flour could affect the gelatinization of the crystalline region. The Tc−To of the flour decreased obviously after dry-heat treatment compared with the control, while that of the modified GRS just had a slight reduction. These changes indicated that dry-heat treatment had a more significant influence on the thermal properties of GRF than GRS, and the difference may be attributed to the existence of the non-starch components such as protein in the flour. The protein in native GRF may have some restrictions during gelatinization and it may make the gelatinization temperature range wider as described by Puncha-arnon and Uttapap [29]. Remarkable reductions were also observed in the endothermic enthalpy change (ΔH) of both GRS and GRF after dry-heat treatment. The ΔH of GRS decreased from 12.09 J/g (GRS) to 11.66 J/g (GRS2 h) and 11.07 J/g (GRS4 h), respectively. In contrast, the flour showed a higher decrease in ΔH from 11.23 J/g (GRF) to 9.74 J/g (GRF2 h) and 8.93 J/g (GRF4 h), respectively. This indicated that dry-heat treatment had a greater influence on the structure of flour than of starch. This finding confirmed the role of non-starch components on properties of modified flours. In addition to rearrangement of starch chains inside starch granules, as suggested by many researchers [30–31] interactions of starch granules and other components in flours during dry-heat treatment would also strengthen the structure of modified flours, as denoted by greater differences in enthalpy change before and after dry-heat treatment of flour samples (1.49–2.10 J/g), as compared to the starch samples (0.43–1.02 J/g). A similar result was reported by Chung et al. [32] who found that adding xanthan to a starch-phosphate mixture prior to dry-heat treatment resulted in reductions in the melting enthalpy. They suggested that this was related to the interactions between starch molecules and xanthan.

**Table 2:** Gelatinization parameters of glutinous rice starch (GRS) and glutinous rice flour (GRF).

Sample	To/°C	Tp/°C	Tc/°C	Tc-To/°C	ΔH/J g^-1^
GRS	59.08±0.10^c^	64.21±0.03^d^	72.09±0.02^e^	13.01±0.30^c^	12.09±0.25^a^
GRS2 h	60.42±0.02^b^	64.98±0.11^c^	73.10±0.12^c^	12.68±0.25^d^	11.66±0.21^b^
GRS4 h	61.59±0.03^a^	67.42±0.12^b^	72.80±0.33^d^	11.21±0.20^e^	11.07±0.20^d^
GRF	57.21±0.06^e^	63.07±0.08^e^	74.49±0.12^a^	17.28±0.42^a^	11.23±0.18^c^
GRF2 h	58.68±0.13^d^	65.09±0.12^c^	73.99±0.07^b^	15.31±0.36^b^	9.74±0.15^e^
GRF4 h	59.16±0.15^c^	67.79±0.04^a^	72.26±0.14^c^	13.10±0.32^c^	8.93±0.10^f^

Values expressed are means ± standard deviations (n = 3). Values of means followed by different lowercase letters in the same column are significantly different (*p* < 0.05).

GRS2 h, 4 h: the glutinous rice starch pastes after dry-heat treatment at 130°C for 2, 4 h; GRF2 h, 4 h: the glutinous rice flour pastes after dry-heat treatment at 130°C for 2, 4 h.

To, Tp, Tc: onset, peak, conclusion temperature; Tc-To: gelatinization temperature range; ΔH: enthalpy change of gelatinization.

### 3.3 Freeze-thaw stability

**Fig 1:**
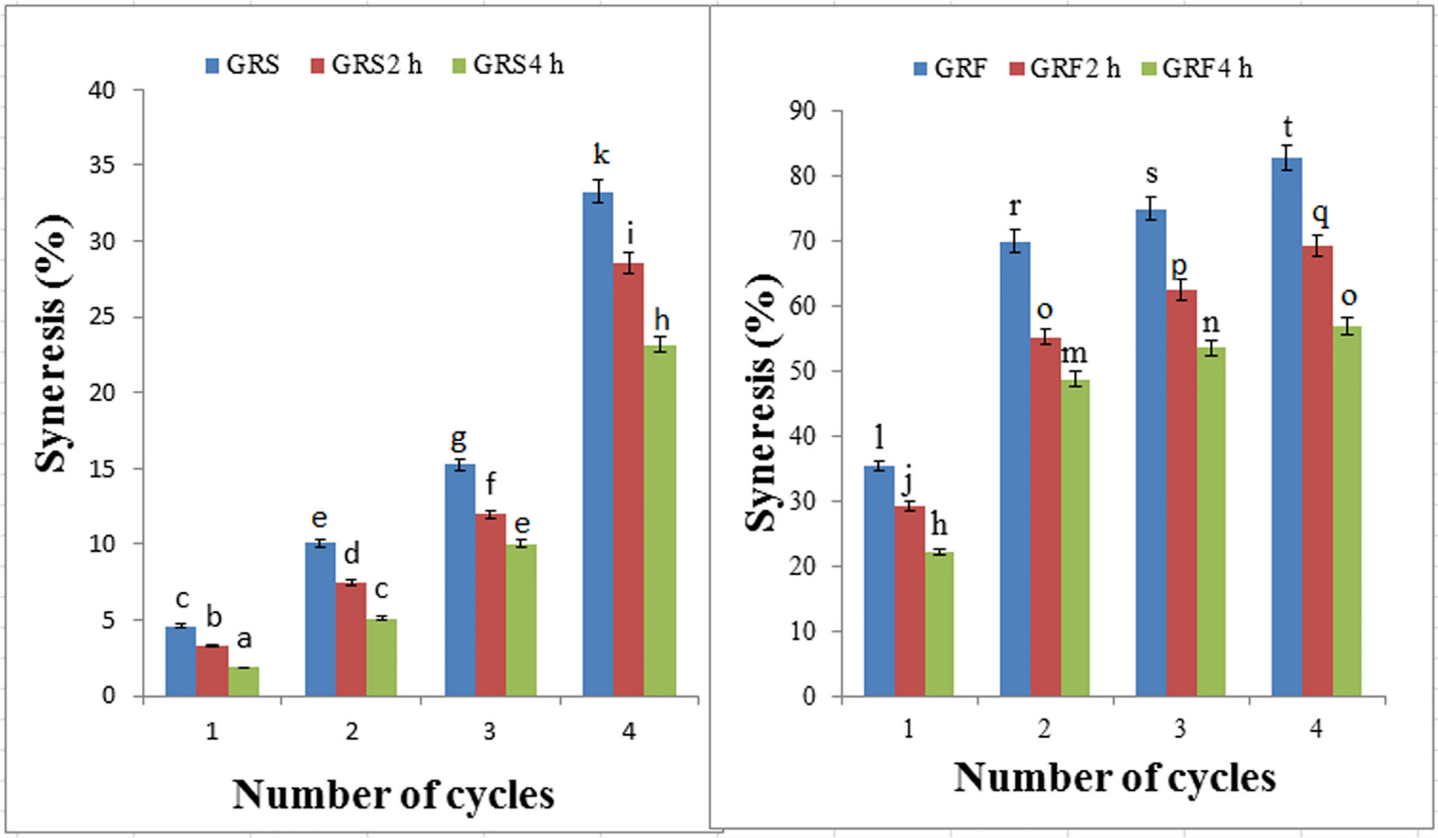
The freeze-thaw stability images of the GRS (A) and GRF (B) before and after dry heating. GRS2 h, 4 h: the glutinous rice starch (GRS) pastes after dry-heat treatment at 130°C for 2, 4 h; GRF2 h, 4 h: the glutinous rice flour (GRF) pastes after dry-heat treatment at 130°C for 2, 4 h. Data represents results from at least three independent experiments and are expressed as mean values ± standard deviations. Different lowercase and capital letters in the same images are significantly different (*p* < 0.05).

Freeze-thaw stability of GRF and GRS gels measured as syneresis, were determined after the 1st, 2nd, 3rd and 4th freeze-thaw cycle. The effect of dry-heat treatment on the amount of syneresis in GRF and GRS gels are presented in Fig 1. The syneresis represents freeze-thaw stability of gels, and the lower values showed better freeze-thaw stability. With increase in the number of freeze-thaw cycle, higher syneresis was observed in the GRF and GRS, probably due to prolong mechanical treatment (centrifugation) that weakened a gel network of starch and starch or protein, which resulting an increase in separating water [33]. Compared to the native GRS from cycle 1 to cycle 4, syneresis of the modified GRS decreased significantly (*p* < 0.05). From cycle 1 to cycle 4, the syneresis in the modified GRS2 h was increased gradually from 3.25% to 7.48%, 11.96% and 28.54%, and then the modified GRS4 h was continuously increased to 1.84%, 5.12%, 10.02%, and 23.18%, respectively. These results indicated that longer dry heating had an improved freeze-thaw stability of GRS.

Compared to the native GRF from cycle 1 to cycle 4, syneresis of modified GRF (Fig 1) was also decreased significantly (*p* < 0.05), suggesting that dry-heat treatment could also enhance the freeze-thaw stability of GRF. Modified GRF2 h showed the syneresis was decreased to 29.26%, 55.23%, 62.42%, and 69.12%, respectively, and then the modified GRF4 h was continuously decreased to 22.17%, 48.69%, 53.47%, and 56.79%, respectively. In contrast to cycle 1, dry-heat treatment had a slight effect on the syneresis of GRF from cycle 2 to cycle 4. In addition, syneresis of native GRF sample was much higher than that of native GRS, indicating that the network structures of native GRF were weaker than those of native GRS. It has been found that interaction of starches with gums resulted in remarkable improvement of syneresis and increases in freeze-thaw stability and an increment of texture quality [34]. Similarly, rice starch gel containing ingredients such as hydrocolloids or protein, which can bind to water molecules, syneresis is reduced [33, 35].

### 3.4 Apparent viscosity versus shear rate

**Fig 2:**
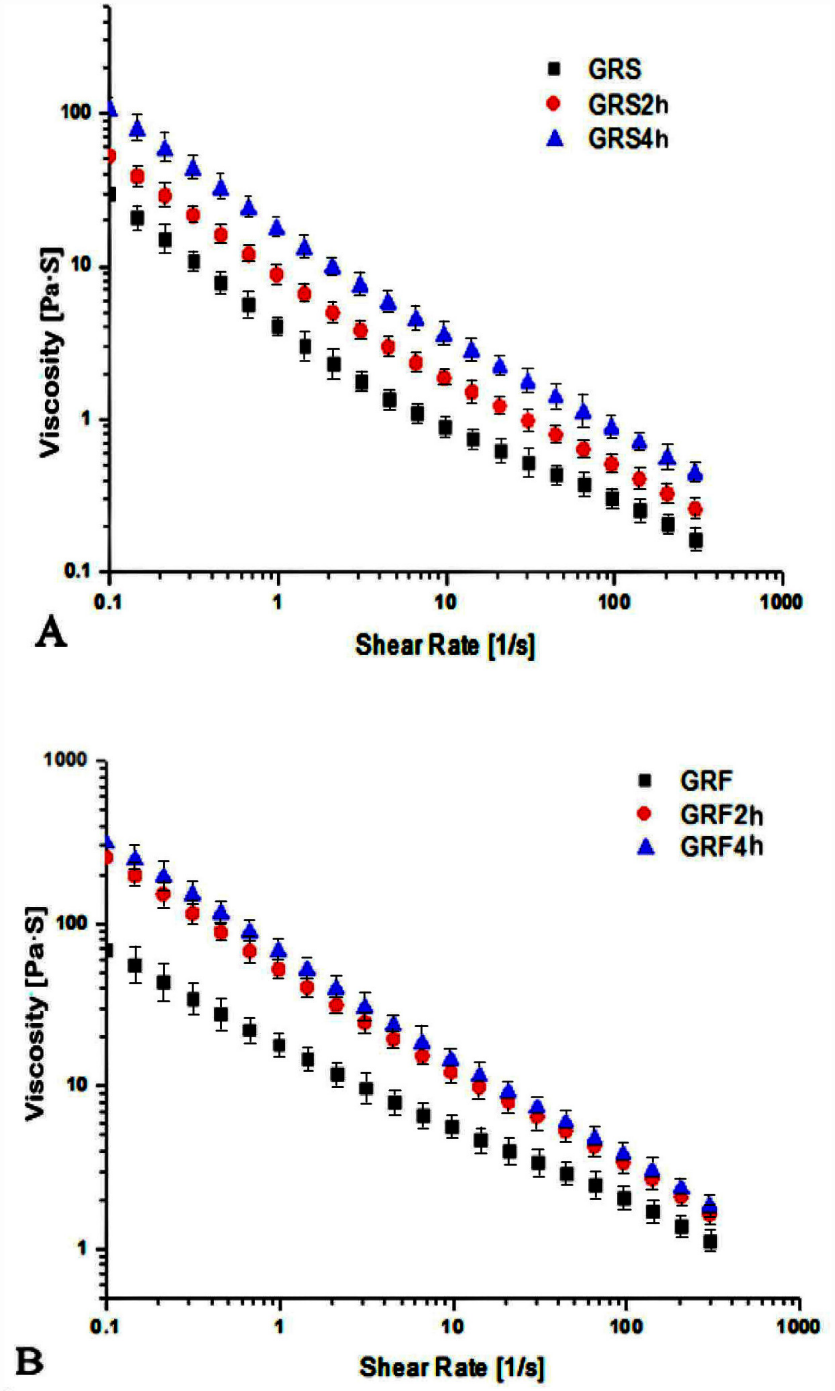
Relationship between apparent viscosity and shearing rate of the GRS (A) and GRF (B) before and after dry heating. GRS2 h, 4 h: the glutinous rice starch (GRS) pastes after dry-heat treatment at 130°C for 2, 4 h; GRF2 h, 4 h: the glutinous rice flour (GRF) pastes after dry-heat treatment at 130°C for 2, 4 h. The data points represent the mean values of three samples and error bars show the standard deviation.

The steady shear flow curves of 12% (w/v) freshly prepared GRF and GRS pastes before and after dry-heat treatment are presented in Fig 2(A) and 2(B). As the shear rate increased, the apparent viscosity of all the GRS pastes decreased gradually, which indicated the system behaved as a pseudoplastic fluid with a shear-thinning property. The reason may be that the gelatinized rice starch pastes formed a stable network structure via the hydrogen bonds, due to the entanglement between starch molecules and the wrapped water molecules. However, increasing of the shear rate would facilitate damage to the network and result in a decrease in apparent viscosity. Compared to the native GRS, the overall apparent viscosity values of modified GRS increased markedly. With the increment of dry heating time from 2 h to 4 h, the apparent viscosity values of GRS continuously increased, indicating that longer dry heating had a much greater impact on the apparent viscosity of GRS. This could be attributed to a stronger interaction between starch molecules through dry-heat treatment, which caused the pastes of GRS to be more shear-resistant and shear-stabilized. Chung et al. [32] also found that the waxy rice starch heated with the mixture of phosphate salts and xanthan exhibited a continuous increase in pasting viscosity. Similar results were reported by Li et al. [13] for waxy rice starches with xanthan, in which the apparent viscosity of the pastes increased after dry heating.

As the shearing rate increased, the apparent viscosity index of GRF samples was also decreased, with the maximum apparent viscosity at 3×10^2^ Pa·s (Fig 2B), which was much higher than that of GRS (1×10^2^ Pa·s) (Fig 2A). At the same dry heating time, the apparent viscosity of GRF was increased to a value more pronounced than that of GRS. At the same shear rate, the apparent viscosity of the dry heated GRF was increased, obviously. However, the apparent viscosity of GRF2 h and GRF4 h changed little. This could be due to the protein in GRF, which plays an important role in their apparent viscosity properties. Lee et al. [36] reported that dry-heat treatment prompted the interaction between cornstarch and soy protein (3%-9%, w/v), as well as increased the pasting viscosity of the starch-soy protein mixture. Qiu et al. [37] reported that the apparent viscosities of waxy cornstarch with soy protein isolate (3%, w/v) increased due to the stronger interactions between starch and protein induced by dry-heat treatment. Furthermore, the interaction of waxy cornstarch with soy protein isolate was more pronounced than that of normal cornstarch with soy protein isolate, mainly due to the higher amylopectin content of the waxy cornstarch. Similar interactions have been reported for starch and gum cross-linking during dry-heat treatments [14, 38]. Our results suggested that dry heated flours could be used instead of starch in rice products, which require a higher viscosity. Furthermore, it contains rich nutrients, such as protein and lipid, as well as avoids the troublesome isolation of starch.

### 3.5 Shear stress versus shear rate

**Fig 3:**
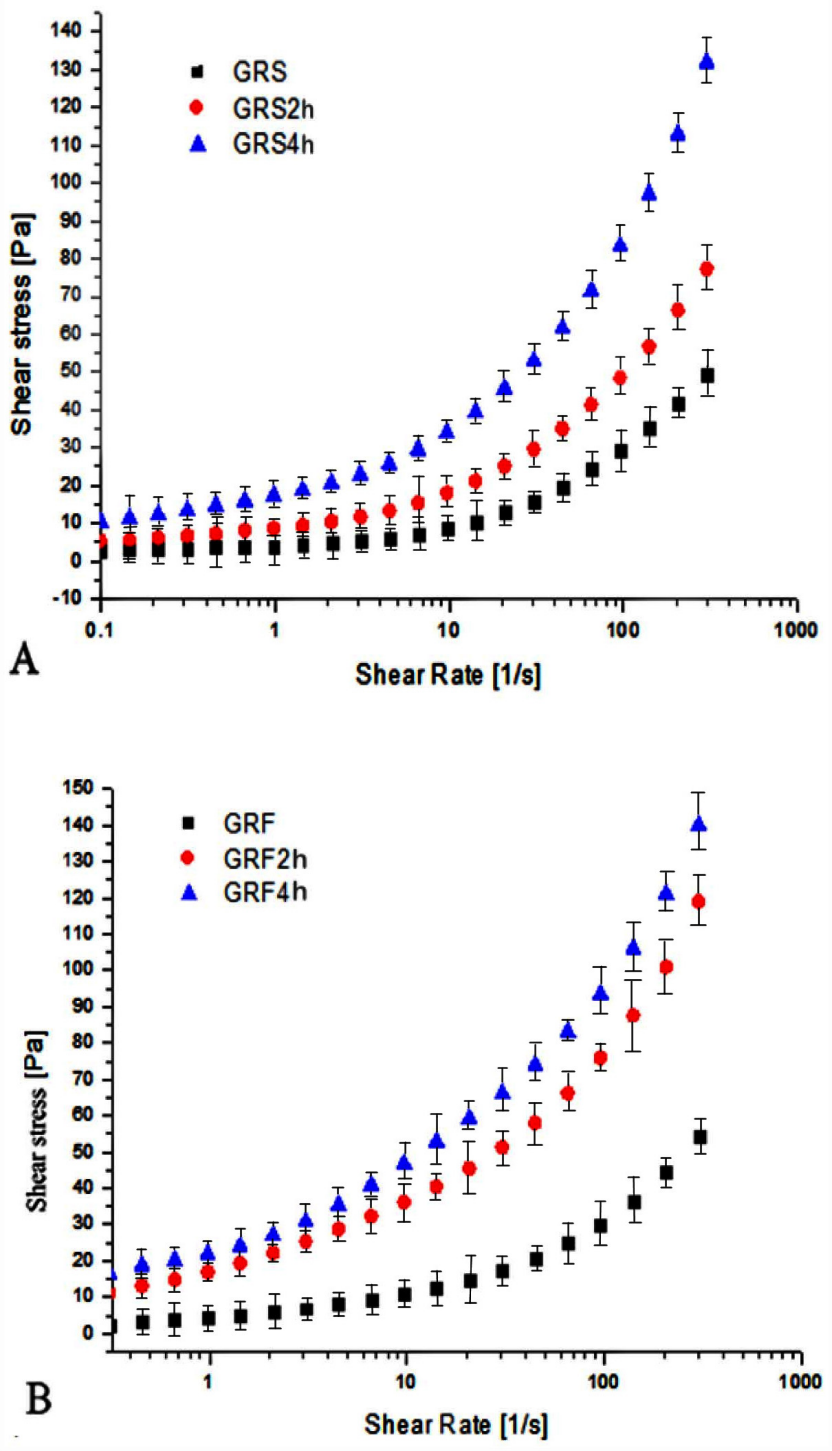
Relationship between shearing stress and shearing rate of GRS (A) and GRF (B) before and after dry heating. GRS2 h, 4 h: the glutinous rice starch (GRS) pastes after dry-heat treatment at 130°C for 2, 4 h; GRF2 h, 4 h: the glutinous rice flour (GRF) pastes after dry-heat treatment at 130°C for 2, 4 h. The data points represent the mean values of three samples and error bars show the standard deviation.

The shear stress versus shear rate rheogram of GRF and GRS before and after dry-heat treatment is presented in Fig 3. The curves of all the samples were non-linear, indicating the systems behaved as non-Newtonian fluids, as well as were considered pseudoplastic fluids [39]. At the same shearing rate, the shearing stress of modified GRS and GRF were apparently larger than that of the control. When the shear rate was 300 s^-1^, the shear stress value of GRS was 50 Pa; However, GRS2 h and GRS4 h increased to 75 Pa and 140 Pa, respectively. Compared to dry heated GRS, GRF showed higher shear stress values after dry-heat treatment, and the shear stress values of GRF, GRF2 h, and GRF4 h were 55 Pa, 120 Pa, and 150 Pa, respectively. The results suggested the structure of modified GRS and GRF were more stable when the dry heating time prolonged. This could be due to the interactions between starches and starches/proteins that would contribute to stabilizing their structures after dry-heat treatment. Our results were in accordance with Sun et al. [40], who studied the effect of microwave-assisted dry heating on cornstarch and waxy cornstarch with xanthan, as well as found the properties of waxy cornstarch to be more affected by heating with xanthan than those of cornstarch.

The parameters of the power law model of Herschel-Bulkley as calculated from graph (Fig 3) are presented in Table 3. A good fit of the experimental data to the rheological equations describing them was indicated by the high values of the R^2^ coefficient (0.97–0.98). The flow behavior index (n) values of all the samples were smaller than 1.0, indicating all the systems exhibited non-Newtonian shear-thinning fluid (pseudoplastic fluid) behaviors under the investigation conditions. The n values of GRF samples were all greater than those of modified GRS. There was no change in the n values of GRS, however, the n values of GRF continuously decreased as the dry heating time increased, indicating the GRF pastes showed stronger pseudoplastic behavior than those of the GRS pastes. Similarly, the n values deceased with increasing of hydroxypropyl starch (HPS) content, meaning the solution showed stronger pseudoplastic behavior with increasing HPS content [41]. Values of the consistency coefficient (K) are a measure of apparent viscosity at the initial stage of shearing [42]. The K values of all modified samples increased, suggesting the starch structure interactions enhanced during dry heating. After dry-heat treatment, the K values of GRS and GRF increased gradually, up to the highest value when the samples were heated for 4 h. The K value of GRS increased from 18.23 Pa·s^n^ to 37.71 Pa·s^n^, and that of GRF increased from 20.77 Pa·s^n^ to 64.34 Pa·s^n^ (Table 3), indicating dry-heat treatment increased interactions between starch molecules in GRS, between starch and protein, or between protein and protein in GRF. The magnitude of K value increased with increasing starch concentration, which was attributed to the strengthened interaction between the particles [38]. Additionally, intermolecular forces of flours were much stronger than those of starches were after dry heating.

**Table 3:** The power law parameters of glutinous rice starch (GRS) and glutinous rice flour (GRF).

Sample	K(Pa.s^n^)	n	R^2^
GRS	18.23±0.85^a^	0.33±0.01^ab^	0.98
GRS2 h	33.11±1.35^c^	0.32±0.01^a^	0.97
GRS4 h	37.71±1.50^d^	0.32±0.01^a^	0.97
GRF	20.77±0.96^b^	0.44±0.01^d^	0.98
GRF2 h	56.57±1.87^e^	0.36±0.01^c^	0.97
GRF4 h	64.34±2.24^f^	0.34±0.00^b^	0.97

Values expressed are means ± standard deviations (n = 3). Values followed by the same letter in the same column are not significantly different (*p* < 0.05).

GRS2 h, 4 h: the glutinous rice starch pastes after dry-heat treatment at 130°C for 2, 4 h; GRF2 h, 4 h: the glutinous rice flour pastes after dry-heat treatment at 130°C for 2, 4 h.

K: consistency coefficient; n: flow behavior index; R^2^: the fitting coefficient.

### 3.6 Dynamic modulus of GRF and GRS before and after dry-heat treatment

**Fig 4:**
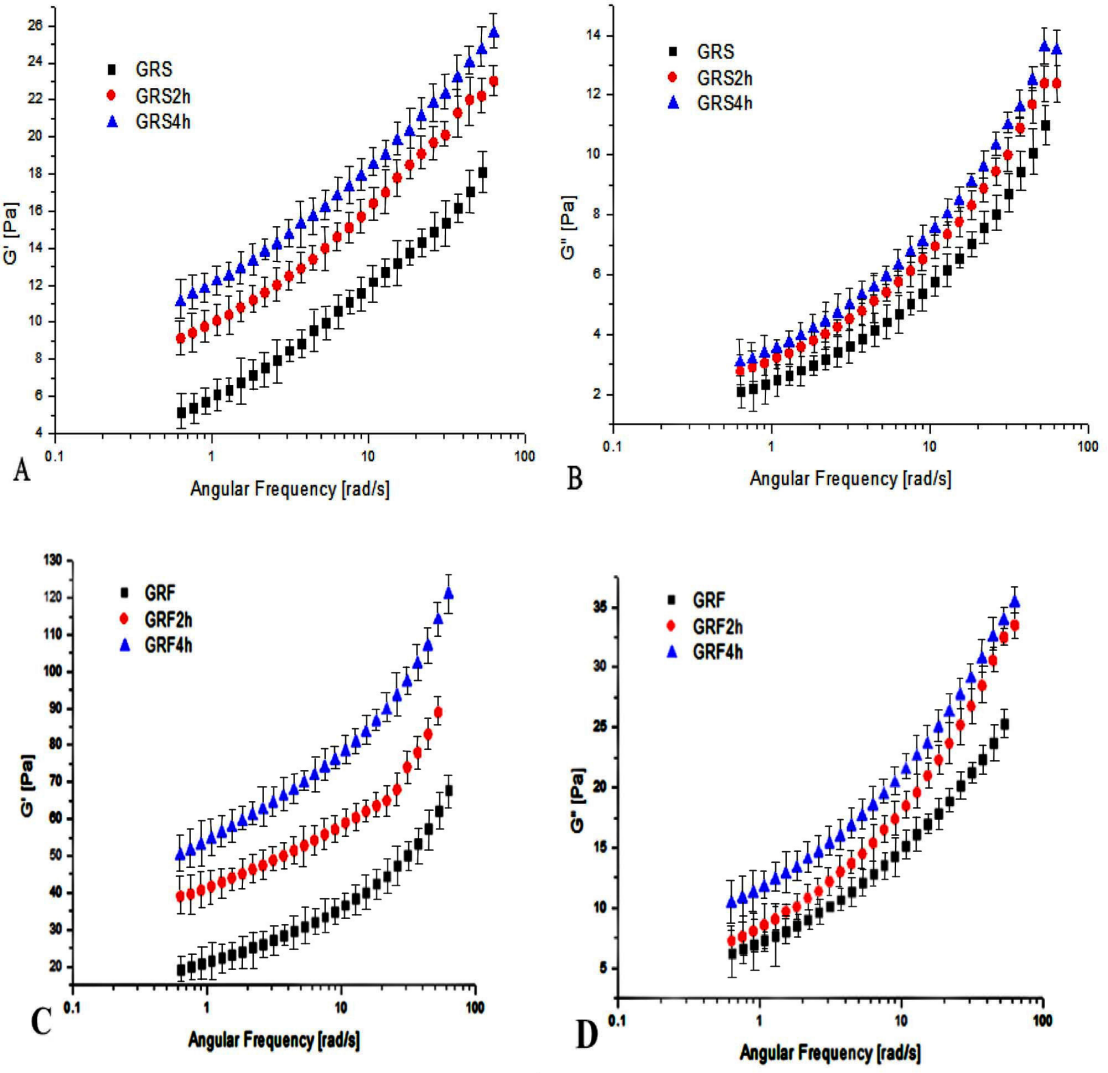
Storage modulus (G') and loss modulus (G") as a function of angular frequency for the GRS (A and B) and GRF (C and D) before and after dry heating. GRS2 h, 4 h: the glutinous rice starch (GRS) pastes after dry-heat treatment at 130°C for 2, 4 h; GRF2 h, 4 h: the glutinous rice flour (GRF) pastes after dry-heat treatment at 130°C for 2, 4 h. The data points represent the mean values of three samples and error bars show the standard deviation.

Fig 4 presents the variations to the storage modulus (G') and loss modulus (G") as a function of frequency (ω) in the GRF and GRS, respectively, before and after dry-heat treatment. The magnitudes of G' and G" increased with an increase in ω, showing a frequency dependency. It was found that the GRS exhibited a weak gel-like behavior [43], as their slopes were positive and the G' values were higher than the G" values at all ω values (0.1–70 rad/s). These results confirmed the viscoelastic nature of the GRS. The G' and G" of native GRS were in the range of 4 to 18 Pa and 2 to 11 Pa, respectively. After dry heat treatment, the G' and G" of GRS4 h increased in the range of 11 to 26 Pa and 2.5 to 13.5 Pa, respectively. This increase in the dynamic moduli can be attributed to an increase in the viscoelastic properties of the dry-heat treatment. The result indicated the dry-heat treatment apparently increased the dynamic modulus (G', G"), influenced the structure, and led to the better viscoelastic capacity of GRS, which was desirable. A similar result was reported by Li et al. [13], who found that both G' and G" increased for the dry heated mixtures of waxy rice starch and xanthan. Thus, the gel-forming ability of the waxy rice starch was strengthened after dry-heat treatment with xanthan.

As depicted in the figure, the dynamic rheological curves of the GRF were consistent with the GRS, but the G' and G" values of GRF were markedly higher than those of GRS were, which suggests dry heating had a more pronounced effect on the elastic properties of the flour than the starch. This increase might be ascribed to the fact that the proteins in GRF produced interactions between them, indicating the increase was more remarkable after dry heating. The G' and G" values of untreated GRF were in the range of 20 to 65 Pa and 6.5 to 25 Pa, respectively. After dry-heat treatment, the G' and G" of GRF samples were in the range of 35 to 125 Pa and 7.5 to 35 Pa, respectively. Furthermore, the magnitudes of the G' and G" values of GRF pastes after dry heating for 4 h were the highest, which indicated the dry heating modification time impacted the G' and G" values of GRF. This trend may have resulted from the interactions of starch and protein during dry-heat treatment [13]. Similarly, Qiu et al. [37] reported that dry-heat treatment resulted in higher G' and G" values of the waxy cornstarch with soy protein isolate, indicating the interactions between starch and soy protein isolate took place after dry heating the starch/soy protein isolate mixture, leading to forming a more viscoelastic gel.

### 3.7 The variation of the tanδ of GRF and GRS with frequency

**Fig 5:**
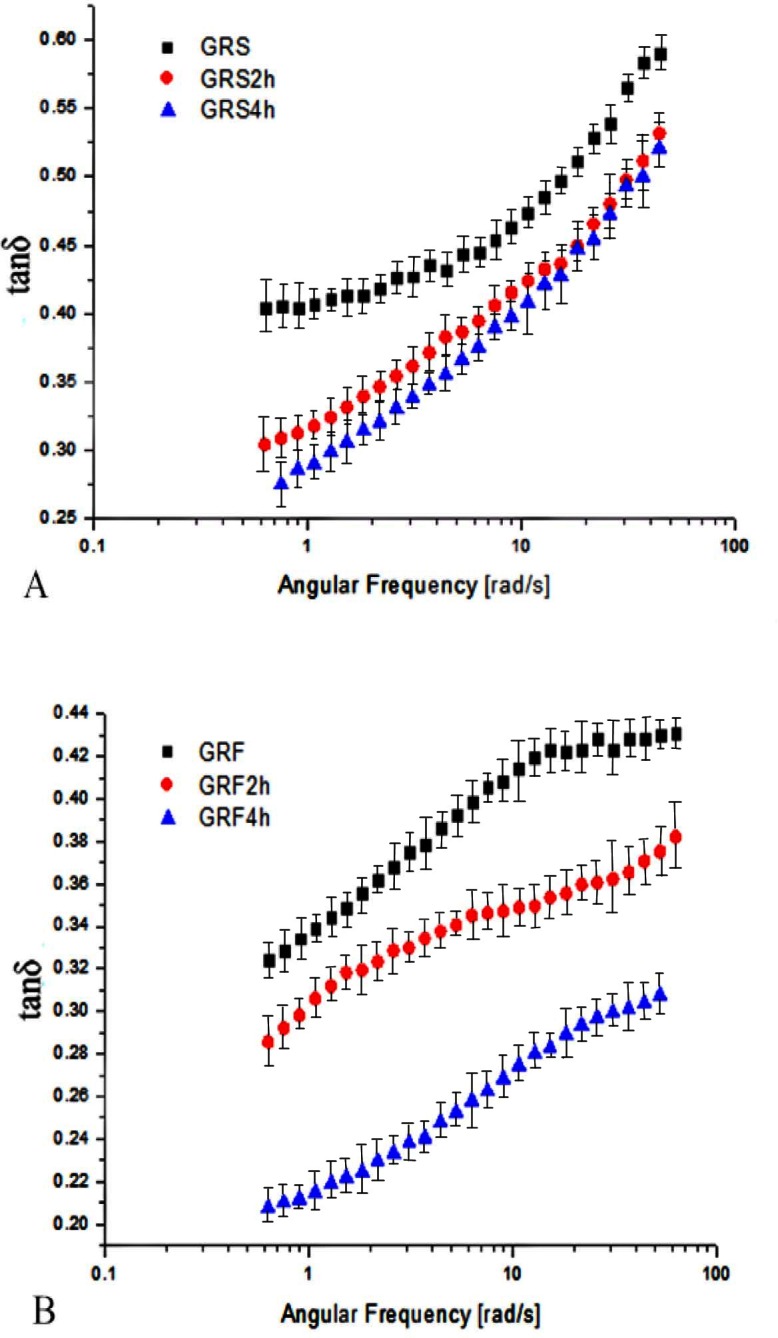
Loss tangent (tanδ = G"/G') as a function of angular frequency for the GRS (A) and GRF (B) before and after dry heating. GRS2 h, 4 h: the glutinous rice starch (GRS) pastes after dry-heat treatment at 130°C for 2, 4 h; GRF2 h, 4 h: the glutinous rice flour (GRF) pastes after dry-heat treatment at 130°C for 2, 4 h. The data points represent the mean values of three samples and error bars show the standard deviation.

The variations of loss tangent (tanδ = G"/ G') as a function of frequency at 25°C are presented in Fig 5. As the frequency increased, the tanδ of GRS and GRF before and after dry-heat treatment increased slightly. The tanδ values of the starches were lower than one, indicating the samples are elastic in nature and have a typical gel network. There was a reduction in the magnitude of the tanδ values of the dry heated samples. The tanδ of GRS was in the range of 0.40 to 0.60, while the tanδ values of GRS2 h and GRS4 h were in the range of 0.28 to 0.55. Similarly, Li et al. [13] also found that the tanδ of the samples decreased when the waxy rice starches and xanthan were dry heated in an electric oven at 130°C for 4 h. However, the tanδ of GRF after dry-heat treatment changed more markedly to be greater than GRF. The tanδ value of GRF was in the range of 0.32 to 0.40, while those of GRF2 h and GRF4 h were in the range of about 0.25 to 0.38. The tanδ value of GRF pastes was obviously lower than that of GRS pastes, which indicated the GRF paste’s structure was stronger and more gel-like than GRS pastes. Similarly, Achayuthakan and Suphantharika [44] reported that the lower the tanδ values of the waxy corn starch/guar gum pates were, the stronger gel behavior the pastes were exhibited. Furthermore, the tanδ of GRF pastes after dry heating for 4 h was more remarkably reduced than GRF2 h, indicating the increased stability of the network was caused by stronger strengthening of the bonds of GRF due to dry heating for a longer period. This could be due to the fact protein or other non-starch ingredients played an important role in the interaction of flours, except starches. Noriko et al. [45] reported that the dry-heat treatment induced improvements in rheological properties in the increasing gel strength of a rather uniform three-dimensional network of interconnected protein strand particles, which were closely packed, and the dry heated egg white proteins resulted in the formation of harder gels.

## Conclusions

Dry-heat treatment played an important role in the rheological, thermal properties and freeze-thaw stability of GRF and GRS. The apparent viscosity of the modified GRF and GRS pastes increased remarkably compared to the control. The G' and G" values of GRF and GRS after dry heating observably increased and the tanδ decreased, indicating the samples after modification exhibited a more gel-like structure. The viscoelastic properties of the GRF after dry heating were enhanced more remarkably than that of GRS samples were, indicating the treatment prompted the interaction between starch and protein, and the GRF paste’s structure was stronger than GRS pastes. The dry heating modification of GRF and GRS can be useful for enhancing their rheological properties. Furthermore, dry heated flour would be used instead of dry heated starch in food products, which require a higher viscoelasticity and gelling, such as sauce, soup, frozen-batter, ice cream and dessert.
